# Supplementary material for: Autistic traits are associated with individual differences in finger tapping: an online study
Source: PeerJ. 2023 May 16;11:e15406. doi: 10.7717/peerj.15406 (PMC10198151; doi:10.7717/peerj.15406)

**Supplementary Materials -** **Differences in finger tapping in adult individuals with autistic traits: An online study**

## Supplementary tables

Table S1

*Exploratory regression results using dominant hand tapping score as the criterion*

| Predictor | *b* | *Confidence interval (LL, UL)* | *t(152)* | *p-value* |
| --- | --- | --- | --- | --- |
| (Intercept) | 81.13** | [73.16, 89.09] | 20.13 | <.001 |
| AQ-10 score | -3.35** | [-5.44, -1.25] | -3.15 | .002 |
| Age | -0.37** | [-0.56, -0.18] | -3.87 | <.001 |
| Gender | 10.42** | [3.84, 17.00] | 3.13 | .002 |
| AQ-10.x Age | 0.07* | [0.01, 0.13] | 2.32 | .033 |
| AQ-10. x Gender | -1.24 | [-3.06, 0.58] | -1.35 | .180 |

*Note.* R^2^ = .233**, 95% CI[.11,.32]. A significant *b*-weight indicates the semi-partial correlation is also significant. *b* represents unstandardized regression weights. *LL* and *UL* indicate the lower and upper limits of a confidence interval, respectively.
* indicates p < .05. ** indicates p < .01.

Table S2

*Exploratory regression results using non-dominant hand tapping score as the criterion*

| Predictor | *b* | *Confidence interval* | *t(152)* | *p-value* |
| --- | --- | --- | --- | --- |
| (Intercept) | 69.62** | [62.35, 76.90] | 18.90 | <.001 |
| AQ-10 score | -1.96* | [-3.87, -0.04] | -2.02 | .045 |
| Age | -0.21* | [-0.38, -0.03] | -3.87 | .021 |
| Gender | 5.90 | [-0.12, 11.91] | 1.94 | .055 |
| AQ-10.x Age | 0.03 | [-0.02, 0.09] | 1.29 | .200 |
| AQ-10 x Gender | -0.49 | [-2.16, 1.17] | -0.59 | .559 |

*Note.* R^2^ = .122**, 95% CI[.02,.20]. A significant *b*-weight indicates the semi-partial correlation is also significant. *b* represents unstandardized regression weights. *LL* and *UL* indicate the lower and upper limits of a confidence interval, respectively.
* indicates p < .05. ** indicates p < .01

## R Packages

The following packages were used in the computation of the results: *corx* (Version 1.0.6.1; Conigrave, 2020), *dplyr* (Version 1.0.7; Wickham et al., 2021), *ggplot2* (Version 3.3.5; Wickham, 2016), *ggridges* (Version 0.5.2; Wilke, 2020), *here* (Version 0.1; Müller, 2017), *interactions* (Version 1.1.5; Long, 2019), *lme4* (Version 1.1.27.1; Bates, Mächler, Bolker, & Walker, 2015), *papaja* (Version 0.1.0.9997; Aust & Barth, 2020), *psych* (Version 2.0.9; Revelle, 2020), *raincloudplots* (Version 0.2.0; Allen M, 2021), *readr* (Version 2.0.0; Wickham & Hester, 2020), *tableone* (Version 0.13.0; Yoshida & Bartel, 2020), and *tidyverse* (Version 1.3.0; Wickham, Averick, et al., 2019.

**R references**:

Allen M, W. K., Poggiali D. (2021). Raincloud plots: A multi-platform tool for robust data visualization [version 2; peer review: 2 approved]. *Wellcome Open Research*, *4*(63). Retrieved from <https://doi.org/10.12688/wellcomeopenres.15191.2>

Aust, F., & Barth, M. (2020). *papaja: Prepare reproducible APA journal articles with R Markdown*. Retrieved from <https://github.com/crsh/papaja>

Bates, D., & Maechler, M. (2019). *Matrix: Sparse and dense matrix classes and methods*. Retrieved from <https://CRAN.R-project.org/package=Matrix>

Conigrave, J. (2020). *Corx: Create and format correlation matrices*. Retrieved from <https://CRAN.R-project.org/package=corx>

Long, J. A. (2019). *Interactions: Comprehensive, user-friendly toolkit for probing interactions*. Retrieved from <https://cran.r-project.org/package=interactions>

Pruim, R., Kaplan, D., & Horton, N. (2020). *MosaicData: Project mosaic data sets*. Retrieved from <https://CRAN.R-project.org/package=mosaicData>

Revelle, W. (2020). *Psych: Procedures for psychological, psychometric, and personality research*. Evanston, Illinois: Northwestern University. Retrieved from <https://CRAN.R-project.org/package=psych>

Wickham, H. (2016). *Ggplot2: Elegant graphics for data analysis*. Springer-Verlag New York. Retrieved from <https://ggplot2.tidyverse.org>

Wickham, H., Averick, M., Bryan, J., Chang, W., McGowan, L. D., François, R., … Yutani, H. (2019). Welcome to the tidyverse. *Journal of Open Source Software*, *4*(43), 1686. <https://doi.org/10.21105/joss.01686>

Wickham, H., François, R., Henry, L., & Müller, K. (2021). *Dplyr: A grammar of data manipulation*. Retrieved from <https://CRAN.R-project.org/package=dplyr>

Wickham, H., & Hester, J. (2020). *Readr: Read rectangular text data*. Retrieved from <https://CRAN.R-project.org/package=readr>

## Information and Consent with demographic questions and screenshots from the Finger Tapping task


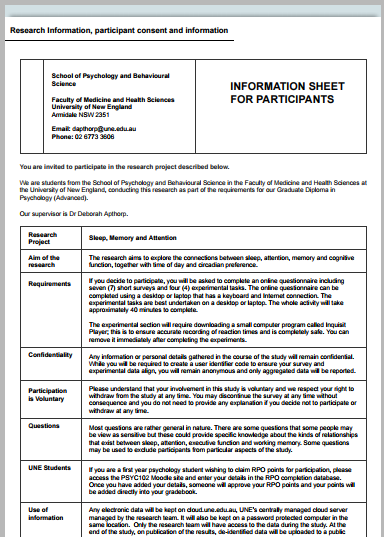


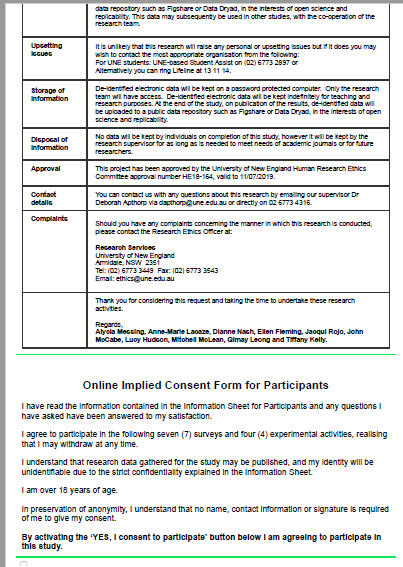


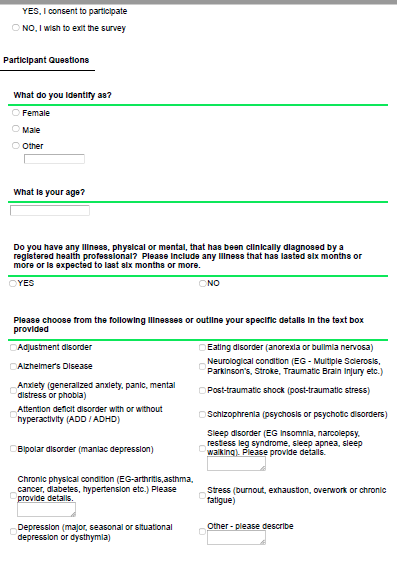


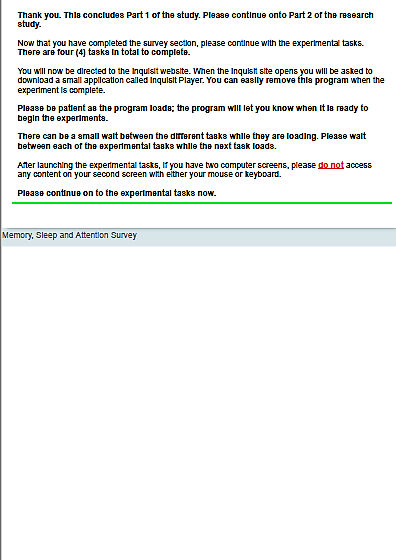


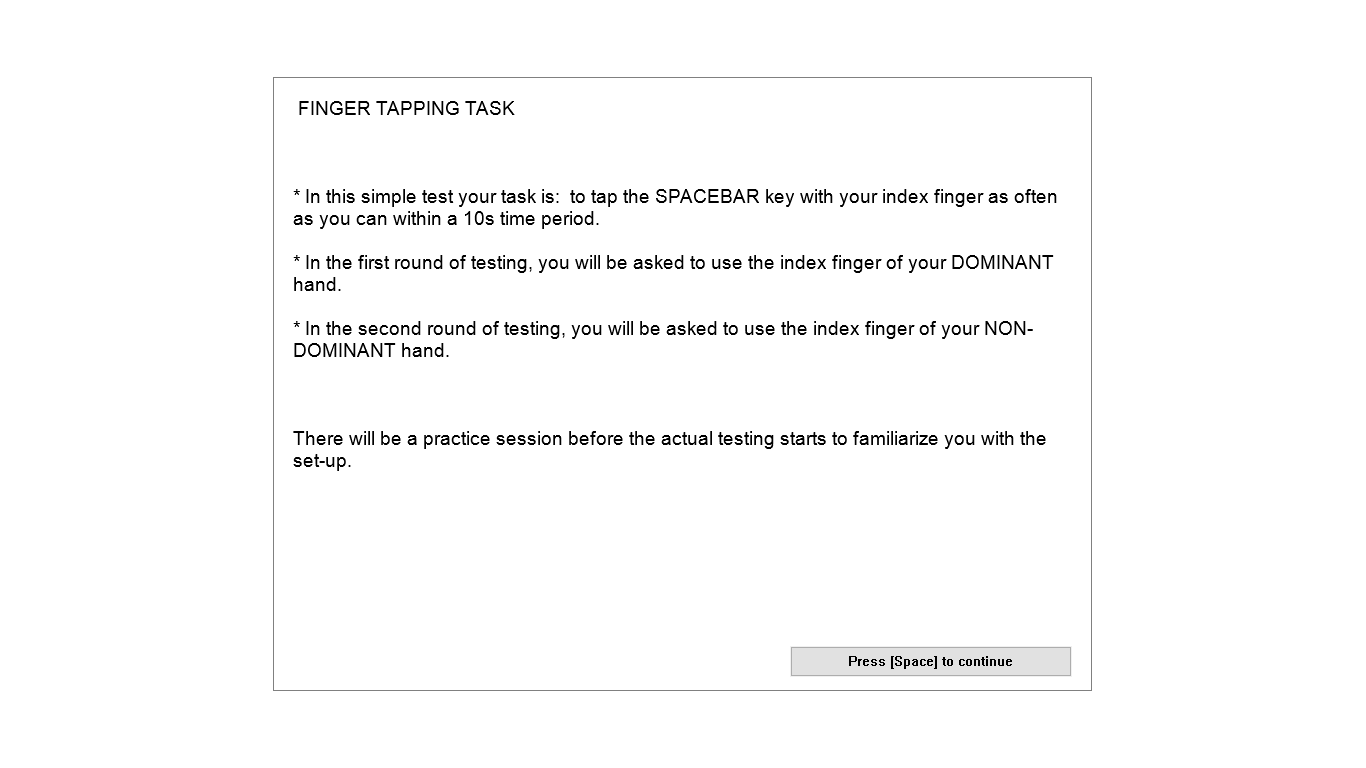

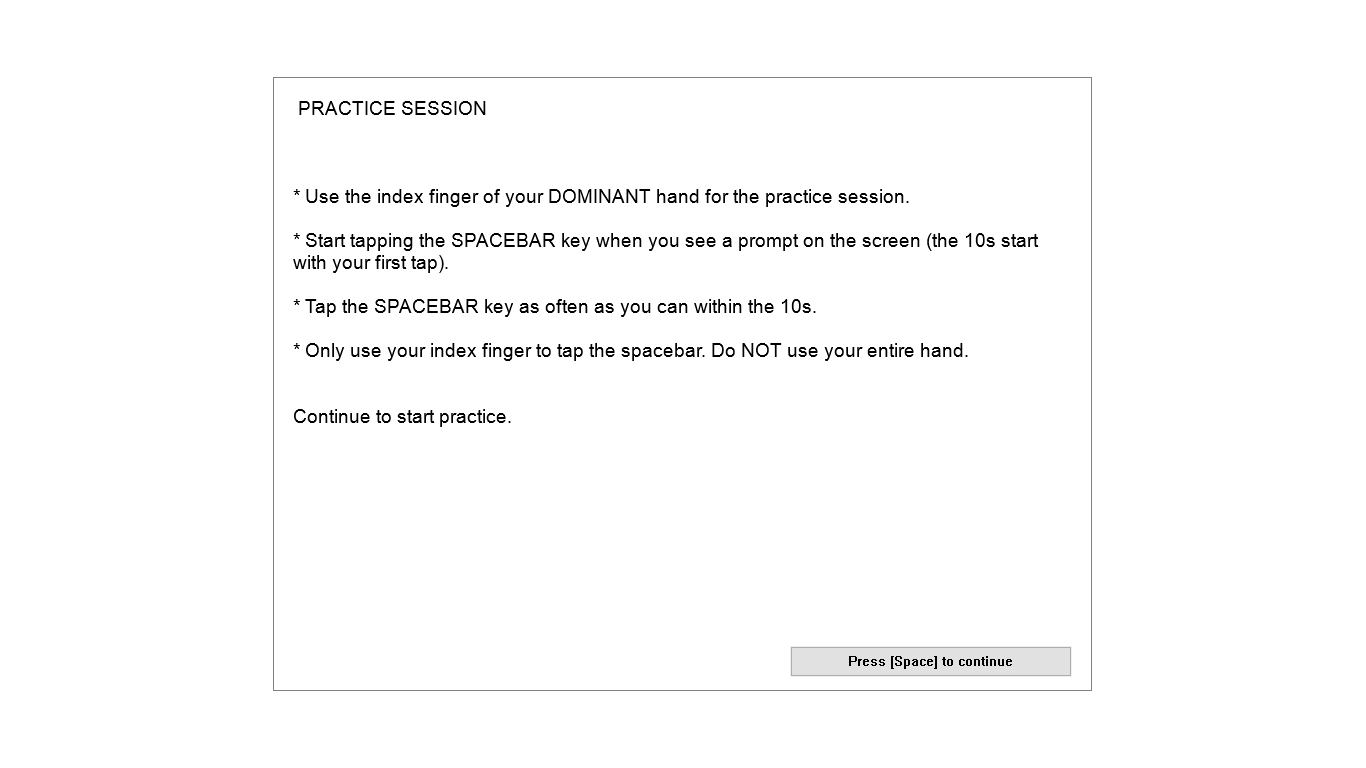

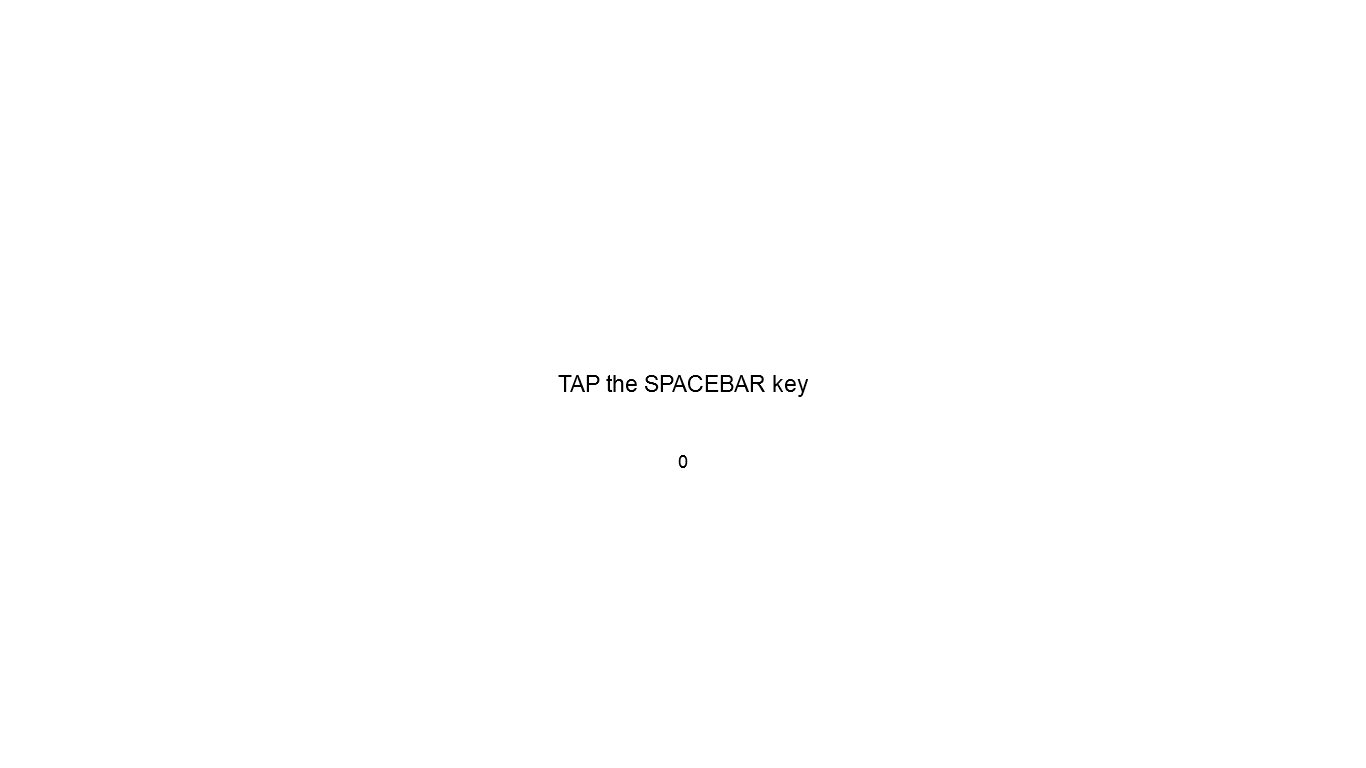

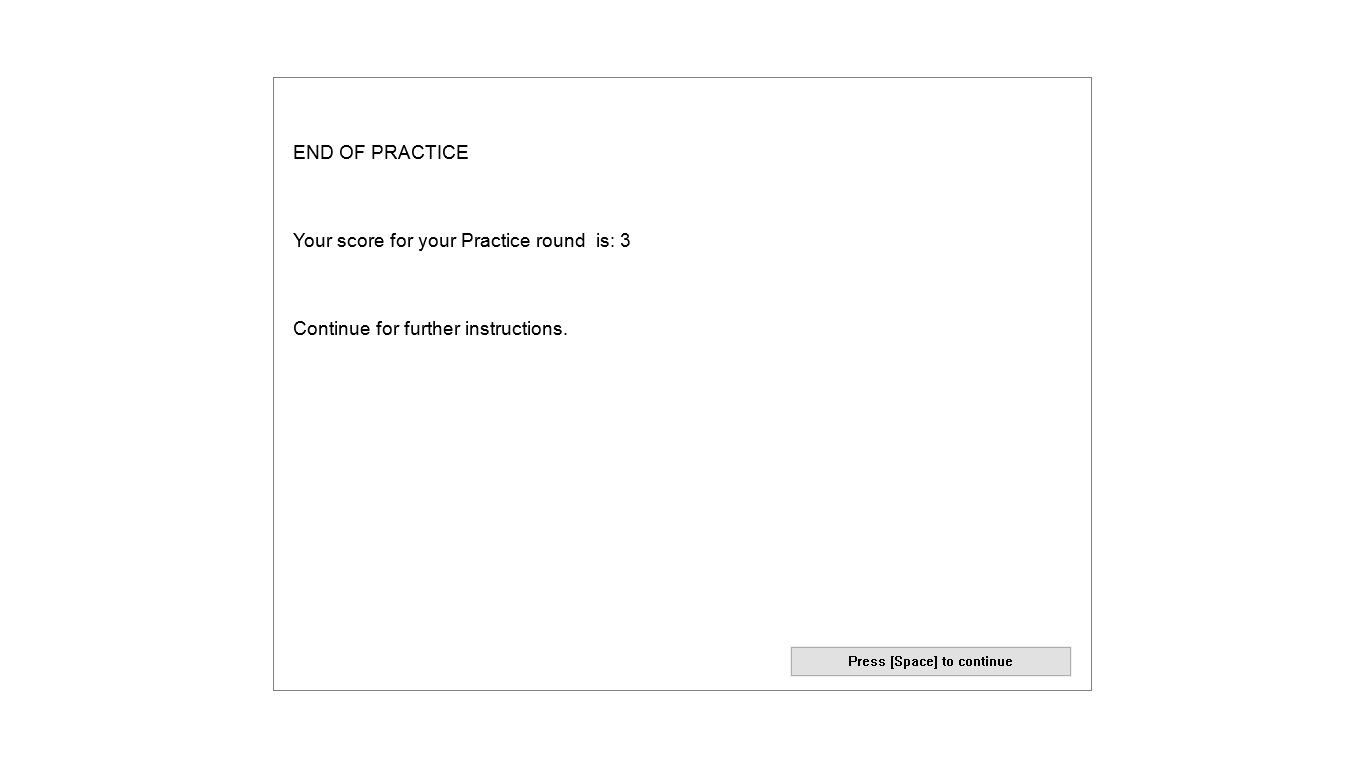

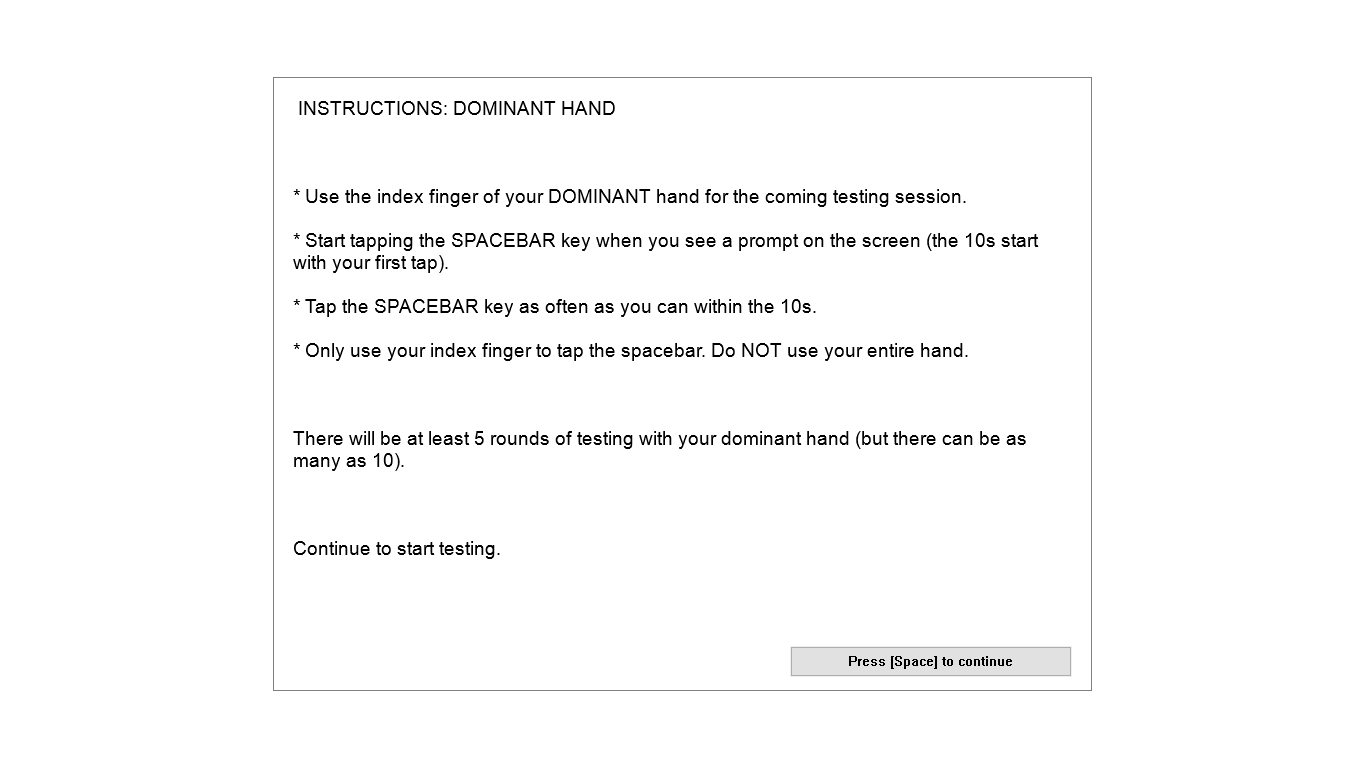

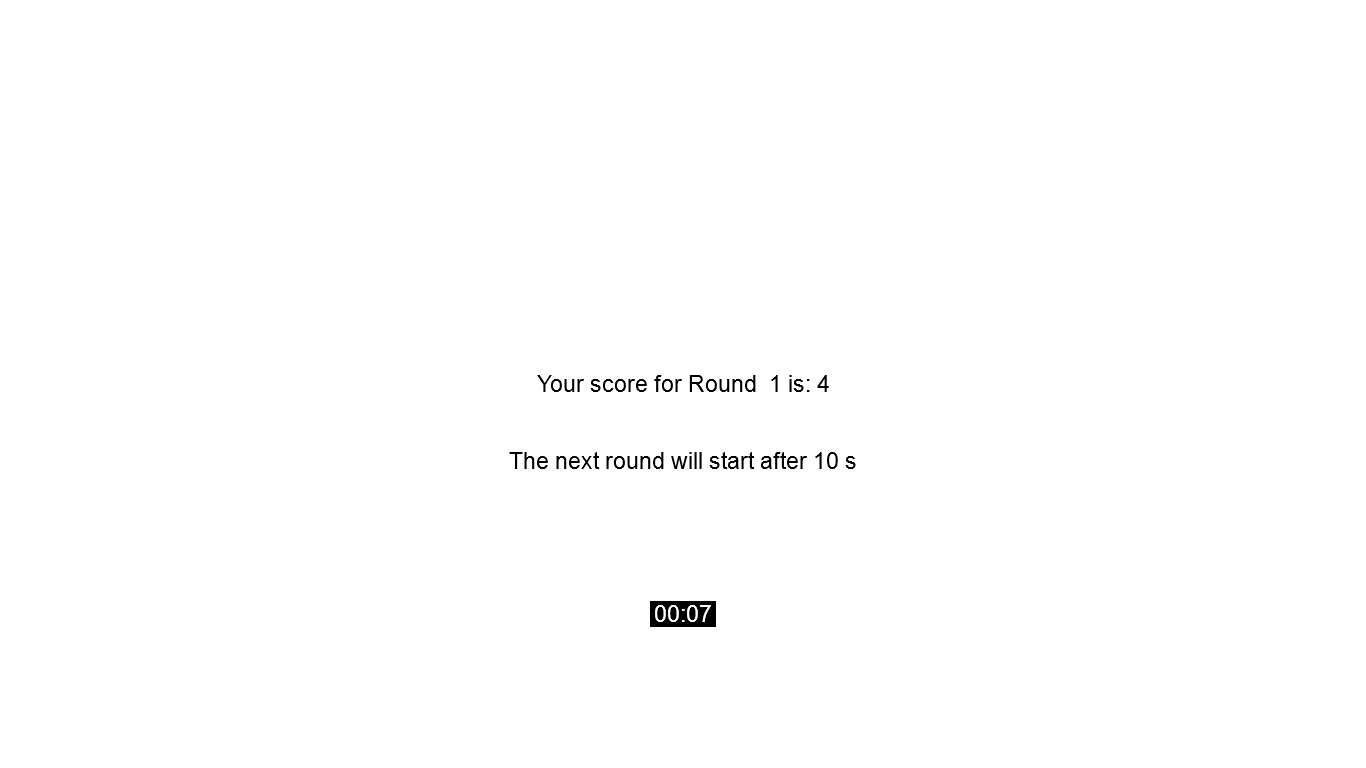

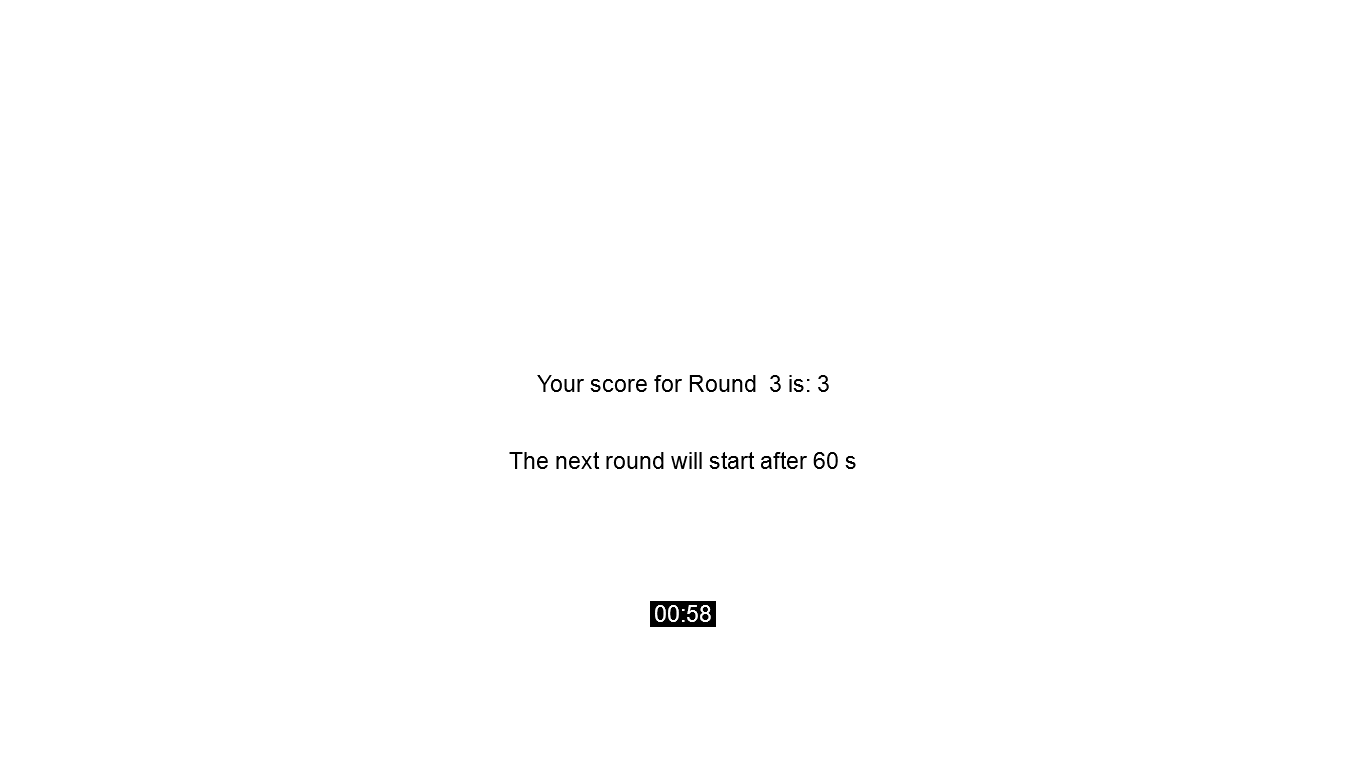

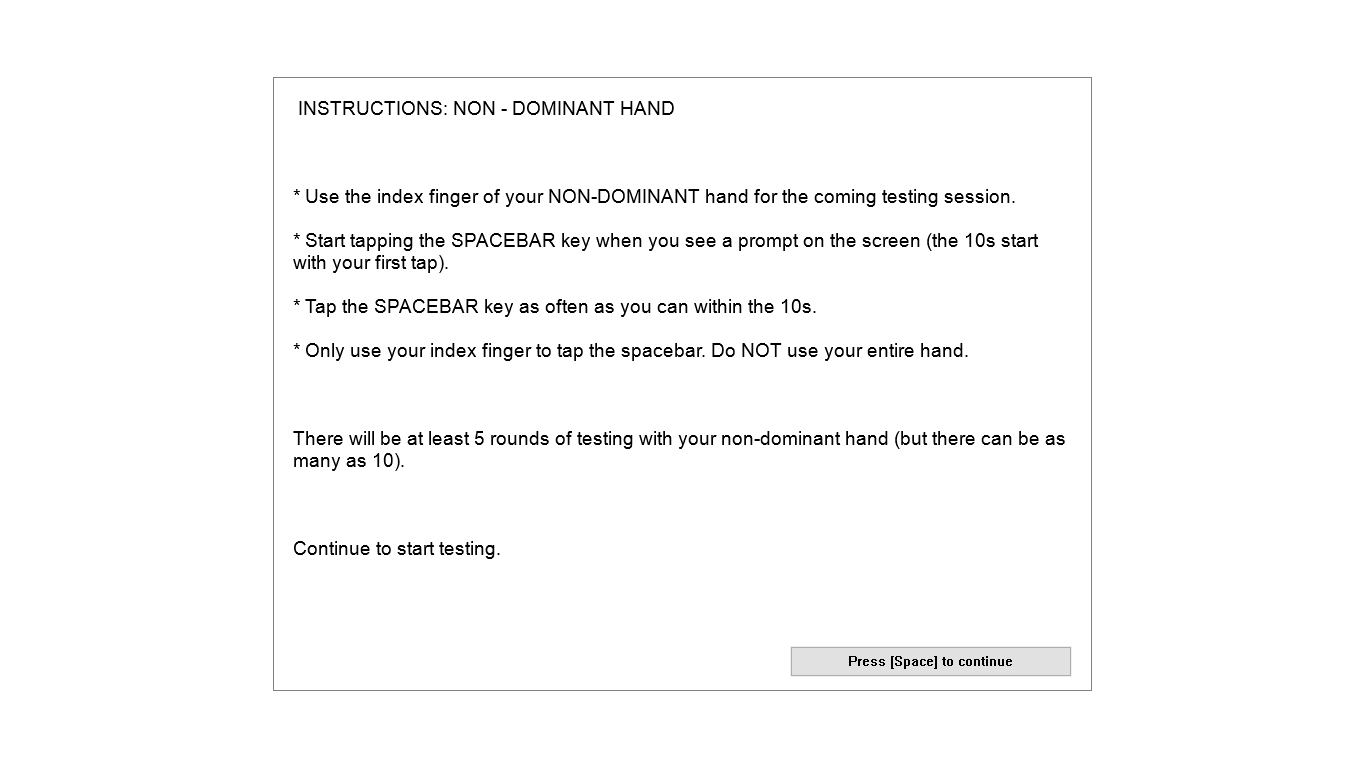

Supplement: Supplemental Information 1 — Supplementary tables for the exploratory regression analyses, as well as screenshots from the online task and information and consent material presented to participants. [file peerj-11-15406-s001.docx]
